# Crystal structure of tris­[μ_2_-bis­(di­phenyl­phosphan­yl)methane-κ^2^ *P*:*P*′]di-μ_3_-bromido-tris­ilver(I) bromide–*N*,*N*′-phenyl­thio­urea (1/1)

**DOI:** 10.1107/S2056989015005150

**Published:** 2015-03-21

**Authors:** Arunpatcha Nimthong-Roldán, Yupa Wattanakanjana, Jintana Rodkeaw

**Affiliations:** a Department of Chemistry, Youngstown State University, 1 University Plaza, 44555 Youngstown, OH, USA; b Department of Chemistry, Faculty of Science, Prince of Songkla University, Hat Yai, Songkhla 90112, Thailand

**Keywords:** crystal structure, silver complex, *N*,*N*′-phenyl­thio­urea

## Abstract

The title complex, [Ag_3_Br_2_(C_25_H_22_P_2_)_3_]Br·C_7_H_8_N_2_S, comprises a trinuclear [Ag_3_Br_2_(C_25_H_22_P_2_)_3_]^+^ unit, a Br^−^ anion and one *N*,*N*′-di­methyl­thio­urea mol­ecule (ptu). Three Ag^I^ ions are linked *via* two μ_3_-bridging Br atoms, leading to a distorted triangular bipyramid with an Ag⋯Ag separation range of 3.1046 (6)–3.3556 (6) Å. The triangular Ag_3_ arrangement is stabilized by six P atoms from three chelating bis­(di­phenyl­phosphan­yl)methane (dppm) ligands. The Ag^I^ ion presents a distorted tetra­hedral coordination geometry. In the crystal, the bromide anion is connected to the ptu mol­ecule through N—H⋯Br hydrogen bonds [graph-set motif *R*_2_^1^(6)]. Each bromide/ptu aggregate links the complex ion *via* C—H⋯S and C—H⋯Br hydrogen bonds, leading to the formation of a three-dimensional network. Two phenyl rings from two dppm ligands were modelled as disordered over two sites.

## Related literature   

For studies of silver(I) complexes with diphosphane ligands, see: Matsumoto *et al.* (2001 ▸); Nicola *et al.* (2005 ▸, 2006 ▸). For their potential applications, see: Song *et al.* (2010 ▸); Sun *et al.* (2011 ▸). For applications of the coordination chemistry of silver(I) complexes with phospho­rus- and sulfur-donor ligands, see: Isab *et al.* (2010 ▸).
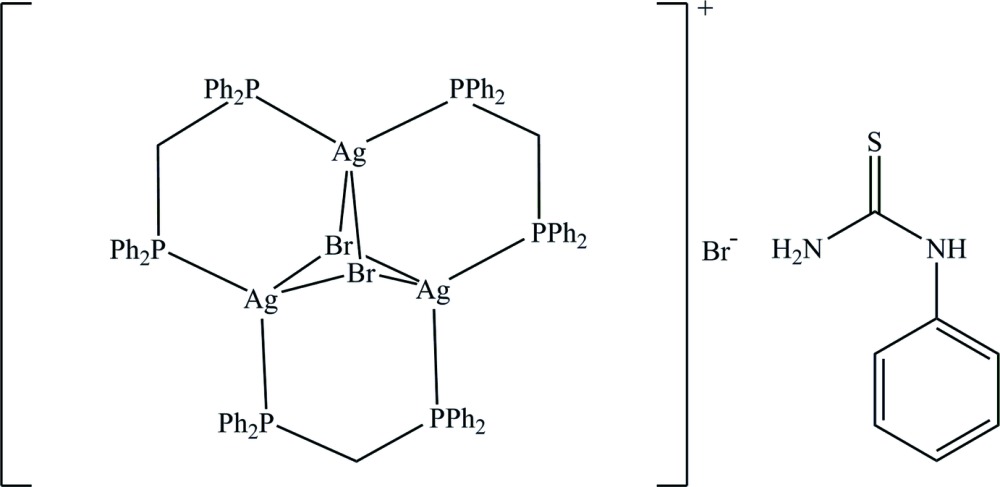


## Experimental   

### Crystal data  

[Ag_3_Br_2_(C_25_H_22_P_2_)_3_]Br·C_7_H_8_N_2_S
*M* *_r_* = 1868.65
Monoclinic, 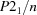

*a* = 10.550 (2) Å
*b* = 28.329 (6) Å
*c* = 25.622 (6) Å
β = 95.859 (4)°
*V* = 7618 (3) Å^3^
*Z* = 4
Mo *K*α radiation
μ = 2.54 mm^−1^
*T* = 100 K
0.22 × 0.15 × 0.07 mm

### Data collection  

Bruker APEXII CCD diffractometer
Absorption correction: multi-scan (*SADABS*; Bruker, 2013 ▸) *T*_min_ = 0.516, *T*_max_ = 0.746
54265 measured reflections
22914 independent reflections
16918 reflections with *I* > 2σ(*I*)
*R*_int_ = 0.045

### Refinement  

*R*[*F* ^2^ > 2σ(*F* ^2^)] = 0.041
*wR*(*F* ^2^) = 0.097
*S* = 0.99
22914 reflections
911 parameters
84 restraints
H-atom parameters constrained
Δρ_max_ = 1.48 e Å^−3^
Δρ_min_ = −0.67 e Å^−3^

### 

Data collection: *APEX2* (Bruker, 2013 ▸); cell refinement: *SAINT* (Bruker, 2013 ▸); data reduction: *SAINT*; program(s) used to solve structure: *SHELXS97* (Sheldrick, 2008 ▸); program(s) used to refine structure: *SHELXL2013* (Sheldrick, 2015 ▸) and *SHELXLE* (Hübschle *et al.*, 2011 ▸); molecular graphics: *Mercury* (Macrae *et al.*, 2008 ▸); software used to prepare material for publication: *publCIF* (Westrip, 2010 ▸).

## Supplementary Material

Crystal structure: contains datablock(s) I, New_Global_Publ_Block. DOI: 10.1107/S2056989015005150/nk2229sup1.cif

Structure factors: contains datablock(s) I. DOI: 10.1107/S2056989015005150/nk2229Isup2.hkl

**Fig. 1.**
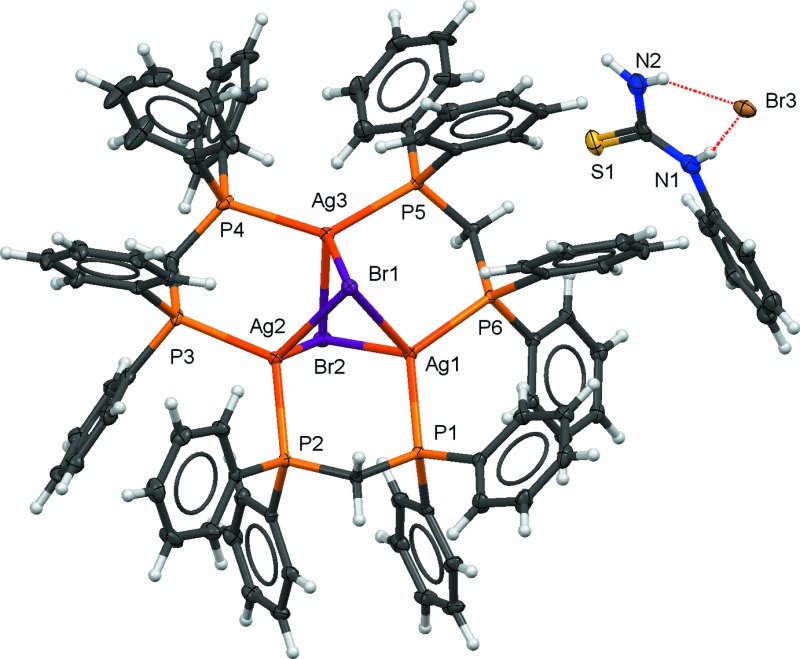
The molecular structure with displacement ellipsoids drawn at the 50% probability level. The minor component of disorder is omitted for clarity. The dashed lines show N—H···Br hydrogen bonds between the ptu and the bromide anion.

. DOI: 10.1107/S2056989015005150/nk2229fig1.tif
The mol­ecular structure with displacement ellipsoids drawn at the 50% probability level. The minor component of disorder is omitted for clarity. The dashed lines show N—H⋯Br hydrogen bonds between the ptu and the bromide anion.

**Fig. 2.**
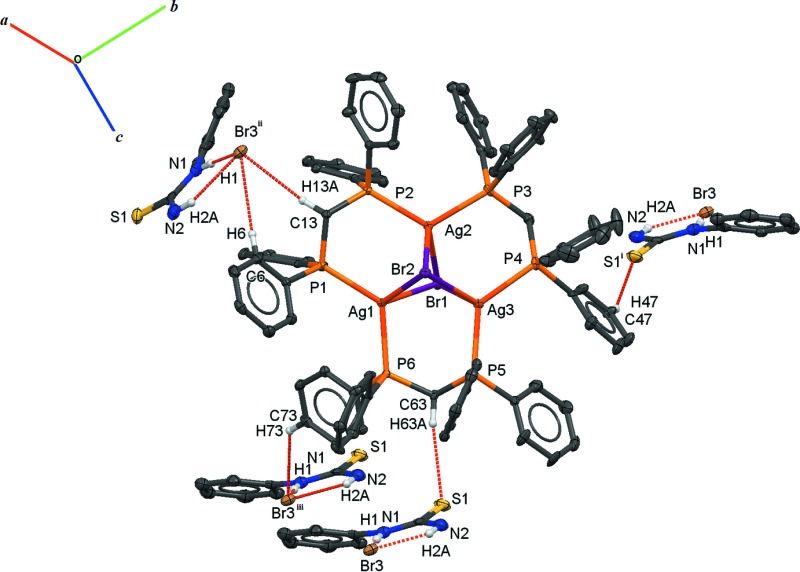
Part of the crystal structure showing intermolecular C—H···S and C—H···Br hydrogen bonds as dashed lines, forming a three-dimensional network.

. DOI: 10.1107/S2056989015005150/nk2229fig2.tif
Part of the crystal structure showing inter­molecular C—H⋯S and C—H⋯Br hydrogen bonds as dashed lines, forming a three-dimensional network.

CCDC reference: 1053790

Additional supporting information:  crystallographic information; 3D view; checkCIF report

## Figures and Tables

**Table 1:** Hydrogen-bond geometry (, )

*D*H*A*	*D*H	H*A*	*D* *A*	*D*H*A*
N1H1Br3	0.88	2.46	3.328(3)	167
N2H2*A*Br3	0.88	2.57	3.390(3)	155
C6H6Br3^i^	0.95	2.93	3.855(3)	166
C13H13*A*Br3^i^	0.99	2.80	3.705(3)	152
C47H47S1^ii^	0.95	2.81	3.645(9)	147
C78H78S1	0.95	2.70	3.259(4)	118

Symmetry codes: (i) 
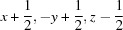
; (ii) 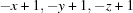.

## supplementary crystallographic information

### S1. Comment

The studies of silver(I) complexes with diphosphane has been receiving more attention (Matsumoto *et al.*, 2001; Nicola *et al.*, 2005; Nicola *et al.*, 2006) because of their potential applications such as show inter­esting luminescence properties (Song *et al.*, 2010; Sun *et al*., 2011). The coordination chemistry of silver(I) complexes with phospho­rus and sulfur donor ligands, on the other hand have been of increasing inter­est due to their potential applications such as anti­microbial activities (Isab *et al.*, 2010). Herein, the title complex was prepared by reacting silver (I) bromide and dppm ligand, followed by the addition of ptu in aceto­nitrile solvent. An unexpexted complex [Ag_3_(C_25_H_22_P_2_)_3_(µ_3_-Br)_2_]^+^ unit was formed in the uncoordinated of ptu.

The title complex comprises of a trinuclear [Ag_3_(C_25_H_22_P_2_)_3_(µ_3_-Br)_2_]^+^ unit, Br anion and one *N*,*N*'-di­methyl­thio­urea molecule (ptu). The bromide anion forms a triple bridge from the both side of the Ag-3 plane leading to distorted triangular bipyramid with an Ag···Ag separation of 3.1046 (6)-3.3556 (6) Å. A triangular Ag_3_ arrangement stabilized by six P atoms from three chelating dppm ligands (Fig.1). The Ag^I^ ion presents a distorted tetra­hedral coordination geometry. In the crystal, The bromide anions is connected to ptu ligand with N—H···Br hydrogen bonds between the ptu NH2 and NHPh moieties and the bromide anion [graph-set motif R^1^_2_(6)] (Fig.1 and Table 1). The dimers are in turn connected with complex ion *via* C—H···S and C—H···Br hydrogen bonds [C63(*sp*^3^)—H63*A*···S1, with H63*A*···S1 = 2.8858 (5) Å, C63(*sp*^3^)···S1 = 3.8147 (7) Å and C63(*sp*^3^)—H63*A*···S1 = 156.604 (4)°; C47(*sp*^2^)—H47···S1^i^, with H47···S1^i^ = 2.8085 (4) Å, C47(*sp*^2^)···S1^i^ = 3.6448 (6) Å and C47(*sp*^2^)—H47···S1^i^ = 147.374 (7)°; C13(*sp*^3^)—H13*A*···Br3^ii^, with H13*A*···Br3^ii^ = 2.8043 (5) Å, C13(*sp*^3^)···Br3^ii^ = 3.7048 (7) Å and C13(*sp*^3^)—H13*A*···Br3^ii^ = 151.555 (6)°; C6(*sp*^2^)—H6···Br3^ii^, with H6···Br3^ii^ = 2.9249 (5) Å, C6(*sp*^2^)···Br3^ii^ = 3.8550 (6) Å and C6(*sp*^2^)—H6···Br3^ii^ = 166.483 (2)°; C73(*sp*^2^)—H73···Br3^iii^, with H73···Br3^iii^ = 3.0296 (5) Å, C73(*sp*^2^)···Br3^ii^ = 3.7706 (5) Å and C73(*sp*^2^)—H73···Br3^iii^ = 135.934 (6)°, symmetry code: (i) 1-x,1-y, 1-z, (ii) 1/2+x, 1/2-y, 1/2+z, (iii) 1+x, y, z] leading to the formation of a three-dimensional network, Fig. 2. In complex, two phenyl rings from two dppm ligands are disordered over two sites with refined occupancies 0.516 (3):0.484 (3).

### S2. Experimental

Bis(di­phenyl­phosphanyl)methane, dppm, (0.1 g, 0.26 mmol) was dissolved in 30 ml of aceto­nitrile at 343 K and then silver(I) bromide, AgBr, (0.05 g, 0.27 mmol) was added. The mixture was stirred for 4 hr and then *N*,*N*′-phenyl­thio­urea, ptu, (0.04 g, 0.26 mmol) was added and the new reaction mixture was heated under reflux for 6 hr during which the precipitate gradually disappeared. The resulting clear solution was filtered and left to evaporate at room temperature. The crystalline complex, which deposited upon standing for several days, was filtered off and dried in vacuo (Mp = 490-492 K).

### S2.1. Refinement

H atoms bonded to C and N atoms were included in calculated positions and were refined with a riding model using distances of 0.95 Å (aryl H), and *U_iso_*(H) = 1.2*U_eq_*(C); 0.99 Å (CH_2_) and *U_iso_*(H) = 1.5*U_eq_*(C); 0.88 Å (NH), and *U_iso_*(H) = 1.2*U_eq_*(N). Two phenyl rings from two dppm ligands are disordered. The ADPs of ipso carbon atoms were constrained to be identical for each disordered pair of phenyl rings. The geometry of the minor moiety of each pair of disordered phenyl rings was restrained to be similar to that of the major moiety (within a standard deviation of 0.02 Angstroms). Carbon atoms of one phenyl ring were restrained with effective standard deviation 0.01 to have the same Uij components. To ensure satisfactory refinement the atoms of each disorder component of the phenyl rings were restrained to lie within a common plane. The overall ratio of the two components of disorder, refined with the same free variable, is 0.516:0.484 (3).

### Crystal data

**Table:**

[Ag_3_Br_2_(C_25_H_22_P_2_)_3_]Br·C_7_H_8_N_2_S	*F*(000) = 3728
*M**_r_* = 1868.65	*D*_x_ = 1.629 Mg m^−^^3^
Monoclinic, *P*2_1_/*n*	Mo *K*α radiation, λ = 0.71073 Å
*a* = 10.550 (2) Å	Cell parameters from 9918 reflections
*b* = 28.329 (6) Å	θ = 2.4–31.0°
*c* = 25.622 (6) Å	µ = 2.54 mm^−^^1^
β = 95.859 (4)°	*T* = 100 K
*V* = 7618 (3) Å^3^	Plate, colourless
*Z* = 4	0.22 × 0.15 × 0.07 mm

### Data collection

**Table:**

Bruker APEXII CCD diffractometer	22914 independent reflections
Radiation source: fine focus sealed tube	16918 reflections with *I* > 2σ(*I*)
Graphite monochromator	*R*_int_ = 0.045
ω and phi scans	θ_max_ = 31.6°, θ_min_ = 1.4°
Absorption correction: multi-scan (*SADABS*; Bruker, 2013)	*h* = −14→15
*T*_min_ = 0.516, *T*_max_ = 0.746	*k* = −41→40
54265 measured reflections	*l* = −37→34

### Refinement

**Table:**

Refinement on *F*^2^	Primary atom site location: structure-invariant direct methods
Least-squares matrix: full	Secondary atom site location: difference Fourier map
*R*[*F*^2^ > 2σ(*F*^2^)] = 0.041	Hydrogen site location: inferred from neighbouring sites
*wR*(*F*^2^) = 0.097	H-atom parameters constrained
*S* = 0.99	*w* = 1/[σ^2^(*F*_o_^2^) + (0.0466*P*)^2^] where *P* = (*F*_o_^2^ + 2*F*_c_^2^)/3
22914 reflections	(Δ/σ)_max_ = 0.002
911 parameters	Δρ_max_ = 1.48 e Å^−^^3^
84 restraints	Δρ_min_ = −0.67 e Å^−^^3^

### Special details

**Table:**

Geometry. All e.s.d.'s (except the e.s.d. in the dihedral angle between two l.s. planes) are estimated using the full covariance matrix. The cell e.s.d.'s are taken into account individually in the estimation of e.s.d.'s in distances, angles and torsion angles; correlations between e.s.d.'s in cell parameters are only used when they are defined by crystal symmetry. An approximate (isotropic) treatment of cell e.s.d.'s is used for estimating e.s.d.'s involving l.s. planes.

### Fractional atomic coordinates and isotropic or equivalent isotropic displacement parameters (Å^2^)

**Table:**

	*x*	*y*	*z*	*U*_iso_*/*U*_eq_	Occ. (<1)
Ag1	0.33769 (2)	0.37216 (2)	0.27599 (2)	0.01259 (5)	
N1	0.1998 (3)	0.22267 (10)	0.52865 (10)	0.0208 (6)	
H1	0.1276	0.2070	0.5232	0.025*	
Ag2	0.32849 (2)	0.46893 (2)	0.21886 (2)	0.01357 (5)	
N2	0.0638 (3)	0.28067 (11)	0.54553 (11)	0.0270 (7)	
H2A	0.0028	0.2596	0.5399	0.032*	
H2B	0.0453	0.3098	0.5538	0.032*	
Ag3	0.23807 (2)	0.46874 (2)	0.33571 (2)	0.01366 (5)	
Br1	0.11287 (3)	0.42417 (2)	0.24594 (2)	0.01306 (6)	
Br2	0.48997 (3)	0.45280 (2)	0.31192 (2)	0.01420 (6)	
Br3	−0.09567 (3)	0.18130 (2)	0.50475 (2)	0.02136 (7)	
S1	0.30352 (10)	0.30847 (3)	0.55186 (4)	0.0294 (2)	
P1	0.39353 (8)	0.33426 (3)	0.19510 (3)	0.01126 (15)	
P2	0.40445 (7)	0.43109 (3)	0.14236 (3)	0.01113 (15)	
P3	0.30916 (8)	0.55483 (3)	0.21908 (3)	0.01422 (16)	
P4	0.22981 (8)	0.55464 (3)	0.32896 (3)	0.01435 (16)	
P5	0.20031 (7)	0.41805 (2)	0.40940 (3)	0.01062 (14)	
P6	0.32377 (7)	0.33211 (2)	0.36017 (3)	0.00953 (14)	
C1	0.3026 (3)	0.28056 (10)	0.17841 (12)	0.0135 (6)	
C2	0.2385 (3)	0.25967 (11)	0.21746 (12)	0.0179 (6)	
H2	0.2538	0.2705	0.2526	0.021*	
C3	0.1523 (3)	0.22307 (11)	0.20549 (13)	0.0218 (7)	
H3	0.1082	0.2094	0.2323	0.026*	
C4	0.1310 (3)	0.20653 (11)	0.15435 (14)	0.0220 (7)	
H4	0.0709	0.1820	0.1459	0.026*	
C5	0.1975 (3)	0.22578 (11)	0.11581 (13)	0.0236 (7)	
H5	0.1850	0.2137	0.0811	0.028*	
C6	0.2824 (3)	0.26262 (11)	0.12729 (12)	0.0189 (7)	
H6	0.3271	0.2757	0.1004	0.023*	
C7	0.5627 (3)	0.32034 (10)	0.19620 (11)	0.0128 (6)	
C8	0.6093 (3)	0.28461 (11)	0.16582 (12)	0.0178 (6)	
H8	0.5517	0.2647	0.1451	0.021*	
C9	0.7398 (3)	0.27816 (12)	0.16592 (12)	0.0222 (7)	
H9	0.7717	0.2533	0.1460	0.027*	
C10	0.8236 (3)	0.30803 (12)	0.19509 (12)	0.0221 (7)	
H10	0.9128	0.3044	0.1941	0.026*	
C11	0.7777 (3)	0.34335 (12)	0.22590 (12)	0.0194 (7)	
H11	0.8354	0.3634	0.2464	0.023*	
C12	0.6478 (3)	0.34914 (11)	0.22651 (11)	0.0165 (6)	
H12	0.6164	0.3730	0.2478	0.020*	
C13	0.3576 (3)	0.36918 (9)	0.13480 (11)	0.0119 (6)	
H13A	0.4023	0.3550	0.1065	0.014*	
H13B	0.2650	0.3675	0.1239	0.014*	
C14	0.5749 (3)	0.43369 (10)	0.13824 (11)	0.0131 (6)	
C15	0.6406 (3)	0.40371 (10)	0.10697 (11)	0.0147 (6)	
H15	0.5949	0.3813	0.0848	0.018*	
C16	0.7720 (3)	0.40657 (11)	0.10827 (12)	0.0187 (7)	
H16	0.8162	0.3864	0.0867	0.022*	
C17	0.8391 (3)	0.43896 (11)	0.14108 (12)	0.0188 (7)	
H17	0.9293	0.4405	0.1423	0.023*	
C18	0.7753 (3)	0.46885 (12)	0.17192 (13)	0.0211 (7)	
H18	0.8215	0.4912	0.1940	0.025*	
C19	0.6439 (3)	0.46623 (10)	0.17076 (12)	0.0175 (6)	
H19	0.6004	0.4867	0.1923	0.021*	
C20	0.3285 (3)	0.45680 (10)	0.08174 (11)	0.0134 (6)	
C21	0.3851 (3)	0.45927 (12)	0.03490 (13)	0.0227 (7)	
H21	0.4668	0.4458	0.0327	0.027*	
C22	0.3213 (4)	0.48155 (13)	−0.00852 (13)	0.0271 (8)	
H22	0.3599	0.4831	−0.0404	0.033*	
C23	0.2030 (3)	0.50133 (12)	−0.00577 (13)	0.0242 (7)	
H23	0.1612	0.5172	−0.0353	0.029*	
C24	0.1450 (3)	0.49800 (12)	0.04033 (13)	0.0215 (7)	
H24	0.0625	0.5109	0.0421	0.026*	
C25	0.2078 (3)	0.47579 (11)	0.08381 (12)	0.0168 (6)	
H25	0.1678	0.4736	0.1153	0.020*	
C26	0.4327 (3)	0.57893 (10)	0.18207 (13)	0.0156 (6)	
C27	0.4095 (3)	0.57758 (11)	0.12688 (13)	0.0197 (7)	
H27	0.3297	0.5666	0.1109	0.024*	
C28	0.5017 (3)	0.59203 (11)	0.09599 (13)	0.0202 (7)	
H28	0.4847	0.5915	0.0589	0.024*	
C29	0.6190 (3)	0.60739 (11)	0.11890 (14)	0.0220 (7)	
H29	0.6823	0.6174	0.0975	0.026*	
C30	0.6441 (3)	0.60825 (12)	0.17276 (13)	0.0224 (7)	
H30	0.7250	0.6184	0.1884	0.027*	
C31	0.5505 (3)	0.59421 (11)	0.20420 (13)	0.0191 (7)	
H31	0.5680	0.5952	0.2413	0.023*	
C32	0.1689 (3)	0.58400 (10)	0.18803 (12)	0.0145 (6)	
C33	0.1700 (3)	0.63239 (11)	0.17634 (14)	0.0215 (7)	
H33	0.2430	0.6508	0.1877	0.026*	
C34	0.0665 (3)	0.65352 (11)	0.14861 (14)	0.0233 (7)	
H34	0.0678	0.6863	0.1407	0.028*	
C35	−0.0399 (3)	0.62635 (12)	0.13241 (14)	0.0226 (7)	
H35	−0.1113	0.6407	0.1131	0.027*	
C36	−0.0436 (3)	0.57915 (11)	0.14380 (13)	0.0187 (7)	
H36	−0.1175	0.5612	0.1326	0.022*	
C37	0.0608 (3)	0.55740 (10)	0.17176 (12)	0.0151 (6)	
H37	0.0583	0.5247	0.1797	0.018*	
C38	0.3365 (3)	0.58099 (10)	0.28477 (12)	0.0172 (6)	
H38A	0.4261	0.5758	0.2991	0.021*	
H38B	0.3215	0.6155	0.2824	0.021*	
C39	0.0748 (3)	0.58239 (11)	0.31259 (12)	0.0181 (6)	
C40	0.0589 (4)	0.63057 (14)	0.3120 (2)	0.0498 (13)	
H40	0.1318	0.6504	0.3164	0.060*	
C41	−0.0612 (4)	0.65071 (15)	0.30531 (19)	0.0478 (13)	
H41	−0.0710	0.6840	0.3057	0.057*	
C42	−0.1668 (4)	0.62162 (15)	0.29795 (15)	0.0342 (9)	
H42	−0.2498	0.6350	0.2937	0.041*	
C43	−0.1522 (4)	0.57369 (15)	0.29676 (17)	0.0365 (10)	
H43	−0.2250	0.5539	0.2911	0.044*	
C44	−0.0307 (3)	0.55371 (13)	0.30385 (14)	0.0251 (8)	
H44	−0.0208	0.5204	0.3026	0.030*	
C45	0.2777 (11)	0.5780 (4)	0.3971 (4)	0.0122 (15)	0.516 (3)
C46	0.3967 (9)	0.5973 (3)	0.4121 (4)	0.0183 (14)	0.516 (3)
H46	0.4587	0.5987	0.3877	0.022*	0.516 (3)
C47	0.4256 (8)	0.6145 (3)	0.4627 (3)	0.0188 (13)	0.516 (3)
H47	0.5071	0.6279	0.4726	0.023*	0.516 (3)
C48	0.3389 (7)	0.6124 (3)	0.4981 (3)	0.0224 (13)	0.516 (3)
H48	0.3585	0.6254	0.5321	0.027*	0.516 (3)
C49	0.2213 (7)	0.5911 (3)	0.4848 (3)	0.0276 (11)	0.516 (3)
H49	0.1623	0.5879	0.5102	0.033*	0.516 (3)
C50	0.1908 (6)	0.5746 (2)	0.4341 (2)	0.0209 (10)	0.516 (3)
H50	0.1096	0.5608	0.4246	0.025*	0.516 (3)
C51	0.2228 (11)	0.4458 (6)	0.4734 (5)	0.0135 (8)	0.516 (3)
C52	0.1633 (6)	0.4293 (2)	0.5154 (2)	0.0249 (11)	0.516 (3)
H52	0.1047	0.4039	0.5104	0.030*	0.516 (3)
C53	0.1885 (7)	0.4497 (3)	0.5653 (3)	0.0307 (12)	0.516 (3)
H53	0.1468	0.4381	0.5939	0.037*	0.516 (3)
C54	0.2734 (9)	0.4864 (5)	0.5727 (5)	0.025 (2)	0.516 (3)
H54	0.2905	0.5002	0.6065	0.030*	0.516 (3)
C55	0.3341 (8)	0.5032 (3)	0.5313 (3)	0.0281 (13)	0.516 (3)
H55	0.3930	0.5286	0.5365	0.034*	0.516 (3)
C56	0.3088 (7)	0.4829 (2)	0.4817 (3)	0.0254 (12)	0.516 (3)
H56	0.3508	0.4946	0.4533	0.030*	0.516 (3)
C45B	0.2865 (11)	0.5844 (5)	0.3875 (4)	0.0122 (15)	0.484 (3)
C46B	0.4167 (10)	0.5857 (3)	0.4039 (4)	0.0183 (14)	0.484 (3)
H46B	0.4758	0.5738	0.3815	0.022*	0.484 (3)
C47B	0.4608 (8)	0.6041 (3)	0.4528 (4)	0.0188 (13)	0.484 (3)
H47B	0.5496	0.6048	0.4638	0.023*	0.484 (3)
C48B	0.3762 (8)	0.6212 (3)	0.4847 (3)	0.0224 (13)	0.484 (3)
H48B	0.4064	0.6351	0.5173	0.027*	0.484 (3)
C49B	0.2443 (7)	0.6183 (3)	0.4699 (3)	0.0276 (11)	0.484 (3)
H49B	0.1856	0.6288	0.4931	0.033*	0.484 (3)
C50B	0.2004 (7)	0.6001 (3)	0.4211 (3)	0.0209 (10)	0.484 (3)
H50B	0.1114	0.5984	0.4107	0.025*	0.484 (3)
C51B	0.2112 (12)	0.4459 (6)	0.4751 (5)	0.0135 (8)	0.484 (3)
C52B	0.1055 (7)	0.4548 (3)	0.5012 (3)	0.0249 (11)	0.484 (3)
H52B	0.0237	0.4451	0.4861	0.030*	0.484 (3)
C53B	0.1177 (8)	0.4779 (3)	0.5496 (3)	0.0307 (12)	0.484 (3)
H53B	0.0443	0.4841	0.5671	0.037*	0.484 (3)
C54B	0.2351 (10)	0.4915 (6)	0.5717 (5)	0.025 (2)	0.484 (3)
H54B	0.2428	0.5073	0.6046	0.030*	0.484 (3)
C55B	0.3418 (9)	0.4827 (3)	0.5471 (3)	0.0281 (13)	0.484 (3)
H55B	0.4234	0.4914	0.5634	0.034*	0.484 (3)
C56B	0.3304 (8)	0.4607 (3)	0.4973 (3)	0.0254 (12)	0.484 (3)
H56B	0.4036	0.4560	0.4792	0.030*	0.484 (3)
C57	0.0437 (3)	0.39098 (10)	0.40417 (11)	0.0139 (6)	
C58	−0.0532 (3)	0.41234 (10)	0.37112 (12)	0.0160 (6)	
H58	−0.0361	0.4402	0.3524	0.019*	
C59	−0.1751 (3)	0.39270 (12)	0.36560 (13)	0.0203 (7)	
H59	−0.2412	0.4074	0.3434	0.024*	
C60	−0.2000 (3)	0.35182 (11)	0.39231 (13)	0.0201 (7)	
H60	−0.2831	0.3385	0.3884	0.024*	
C61	−0.1038 (3)	0.33040 (11)	0.42470 (12)	0.0176 (6)	
H61	−0.1208	0.3021	0.4426	0.021*	
C62	0.0169 (3)	0.34995 (10)	0.43120 (11)	0.0142 (6)	
H62	0.0819	0.3354	0.4541	0.017*	
C63	0.3137 (3)	0.36871 (10)	0.41920 (11)	0.0124 (6)	
H63A	0.2883	0.3484	0.4478	0.015*	
H63B	0.3993	0.3816	0.4306	0.015*	
C64	0.1905 (3)	0.29088 (10)	0.35797 (11)	0.0099 (5)	
C65	0.0845 (3)	0.29975 (10)	0.32184 (11)	0.0131 (6)	
H65	0.0826	0.3271	0.3003	0.016*	
C66	−0.0178 (3)	0.26867 (11)	0.31741 (12)	0.0159 (6)	
H66	−0.0896	0.2748	0.2929	0.019*	
C67	−0.0153 (3)	0.22896 (11)	0.34855 (12)	0.0177 (6)	
H67	−0.0852	0.2077	0.3452	0.021*	
C68	0.0886 (3)	0.21991 (11)	0.38471 (13)	0.0196 (7)	
H68	0.0895	0.1926	0.4063	0.024*	
C69	0.1915 (3)	0.25077 (10)	0.38943 (12)	0.0164 (6)	
H69	0.2627	0.2445	0.4142	0.020*	
C70	0.4627 (3)	0.29512 (10)	0.37822 (11)	0.0110 (5)	
C71	0.5029 (3)	0.28114 (10)	0.42952 (11)	0.0141 (6)	
H71	0.4571	0.2913	0.4575	0.017*	
C72	0.6092 (3)	0.25244 (10)	0.43993 (12)	0.0151 (6)	
H72	0.6358	0.2433	0.4750	0.018*	
C73	0.6766 (3)	0.23708 (10)	0.39950 (12)	0.0161 (6)	
H73	0.7496	0.2176	0.4068	0.019*	
C74	0.6371 (3)	0.25031 (10)	0.34796 (12)	0.0159 (6)	
H74	0.6821	0.2394	0.3200	0.019*	
C75	0.5318 (3)	0.27945 (10)	0.33759 (12)	0.0135 (6)	
H75	0.5062	0.2889	0.3025	0.016*	
C76	0.1869 (3)	0.26821 (12)	0.54145 (12)	0.0227 (7)	
C77	0.3106 (3)	0.19549 (11)	0.52243 (12)	0.0199 (7)	
C78	0.4293 (3)	0.20346 (12)	0.55037 (13)	0.0242 (7)	
H78	0.4413	0.2292	0.5741	0.029*	
C79	0.5292 (4)	0.17338 (13)	0.54304 (15)	0.0288 (8)	
H79	0.6102	0.1790	0.5618	0.035*	
C80	0.5148 (4)	0.13535 (13)	0.50923 (15)	0.0293 (8)	
H80	0.5845	0.1151	0.5045	0.035*	
C81	0.3955 (4)	0.12751 (12)	0.48229 (14)	0.0287 (8)	
H81	0.3834	0.1014	0.4591	0.034*	
C82	0.2940 (3)	0.15714 (12)	0.48869 (13)	0.0228 (7)	
H82	0.2131	0.1513	0.4700	0.027*	

### Atomic displacement parameters (Å^2^)

**Table:**

	*U*^11^	*U*^22^	*U*^33^	*U*^12^	*U*^13^	*U*^23^
Ag1	0.01728 (12)	0.01151 (10)	0.00935 (10)	0.00142 (8)	0.00309 (8)	0.00115 (7)
N1	0.0172 (14)	0.0257 (14)	0.0193 (14)	−0.0012 (11)	0.0017 (12)	0.0029 (11)
Ag2	0.01765 (12)	0.01044 (10)	0.01319 (11)	0.00094 (8)	0.00432 (9)	0.00010 (8)
N2	0.0298 (17)	0.0256 (15)	0.0261 (16)	0.0045 (13)	0.0058 (13)	0.0087 (12)
Ag3	0.01611 (12)	0.01252 (10)	0.01280 (11)	−0.00024 (8)	0.00365 (9)	0.00067 (8)
Br1	0.01077 (14)	0.01522 (13)	0.01283 (14)	−0.00082 (11)	−0.00048 (11)	0.00105 (10)
Br2	0.01078 (14)	0.01633 (14)	0.01521 (15)	−0.00028 (11)	−0.00002 (11)	−0.00229 (11)
Br3	0.01916 (17)	0.03082 (18)	0.01429 (15)	0.00201 (13)	0.00257 (13)	0.00306 (12)
S1	0.0366 (6)	0.0236 (4)	0.0284 (5)	−0.0072 (4)	0.0054 (4)	0.0083 (4)
P1	0.0146 (4)	0.0101 (3)	0.0090 (3)	0.0009 (3)	0.0012 (3)	0.0001 (3)
P2	0.0130 (4)	0.0104 (3)	0.0100 (4)	0.0014 (3)	0.0017 (3)	0.0011 (3)
P3	0.0124 (4)	0.0086 (3)	0.0217 (4)	0.0009 (3)	0.0020 (3)	0.0019 (3)
P4	0.0127 (4)	0.0116 (3)	0.0187 (4)	0.0003 (3)	0.0009 (3)	−0.0042 (3)
P5	0.0112 (4)	0.0100 (3)	0.0107 (4)	0.0000 (3)	0.0013 (3)	−0.0006 (3)
P6	0.0097 (4)	0.0099 (3)	0.0090 (3)	0.0007 (3)	0.0013 (3)	0.0010 (3)
C1	0.0160 (16)	0.0104 (13)	0.0142 (15)	0.0007 (11)	0.0014 (12)	0.0000 (10)
C2	0.0219 (17)	0.0161 (14)	0.0161 (16)	−0.0004 (12)	0.0042 (13)	−0.0017 (11)
C3	0.0237 (18)	0.0192 (15)	0.0240 (18)	−0.0010 (13)	0.0103 (15)	0.0010 (13)
C4	0.0195 (17)	0.0125 (14)	0.033 (2)	−0.0022 (12)	−0.0014 (15)	0.0011 (13)
C5	0.035 (2)	0.0157 (15)	0.0188 (17)	−0.0007 (14)	−0.0041 (15)	−0.0019 (12)
C6	0.0271 (18)	0.0169 (14)	0.0122 (15)	−0.0023 (13)	−0.0005 (13)	0.0004 (11)
C7	0.0149 (15)	0.0165 (14)	0.0073 (13)	0.0033 (11)	0.0019 (11)	0.0026 (10)
C8	0.0237 (18)	0.0180 (14)	0.0113 (15)	0.0040 (13)	0.0006 (13)	−0.0025 (11)
C9	0.0265 (19)	0.0274 (17)	0.0135 (16)	0.0124 (14)	0.0056 (14)	−0.0004 (13)
C10	0.0152 (17)	0.0358 (19)	0.0156 (16)	0.0099 (14)	0.0031 (13)	0.0065 (14)
C11	0.0192 (17)	0.0252 (16)	0.0135 (15)	0.0009 (13)	−0.0003 (13)	0.0037 (12)
C12	0.0217 (17)	0.0171 (14)	0.0105 (14)	0.0034 (12)	0.0008 (12)	0.0001 (11)
C13	0.0140 (15)	0.0112 (13)	0.0102 (14)	0.0009 (11)	0.0000 (11)	0.0011 (10)
C14	0.0154 (15)	0.0113 (13)	0.0129 (14)	0.0016 (11)	0.0028 (12)	0.0032 (10)
C15	0.0159 (16)	0.0168 (14)	0.0111 (14)	0.0007 (12)	0.0005 (12)	0.0003 (11)
C16	0.0177 (17)	0.0233 (16)	0.0159 (16)	0.0034 (13)	0.0060 (13)	0.0014 (12)
C17	0.0112 (16)	0.0270 (17)	0.0180 (16)	0.0002 (13)	0.0005 (13)	0.0043 (13)
C18	0.0200 (18)	0.0237 (16)	0.0194 (17)	−0.0071 (13)	0.0007 (14)	−0.0022 (13)
C19	0.0189 (17)	0.0141 (14)	0.0196 (16)	−0.0021 (12)	0.0020 (13)	−0.0014 (12)
C20	0.0180 (16)	0.0107 (13)	0.0111 (14)	0.0012 (11)	−0.0004 (12)	0.0011 (10)
C21	0.0205 (18)	0.0307 (18)	0.0175 (17)	0.0090 (14)	0.0052 (14)	0.0076 (13)
C22	0.028 (2)	0.040 (2)	0.0141 (17)	0.0044 (16)	0.0052 (15)	0.0104 (14)
C23	0.0246 (19)	0.0271 (17)	0.0193 (17)	0.0013 (14)	−0.0052 (14)	0.0094 (13)
C24	0.0153 (17)	0.0241 (16)	0.0241 (18)	0.0050 (13)	−0.0027 (14)	0.0014 (13)
C25	0.0171 (16)	0.0194 (15)	0.0138 (15)	0.0010 (12)	0.0015 (12)	0.0009 (11)
C26	0.0107 (15)	0.0131 (14)	0.0232 (17)	0.0026 (11)	0.0031 (12)	0.0029 (11)
C27	0.0186 (17)	0.0177 (15)	0.0224 (17)	−0.0003 (13)	−0.0001 (14)	0.0005 (12)
C28	0.0201 (17)	0.0209 (15)	0.0195 (17)	0.0019 (13)	0.0006 (14)	0.0025 (12)
C29	0.0156 (17)	0.0216 (16)	0.0300 (19)	0.0020 (13)	0.0077 (14)	0.0044 (13)
C30	0.0141 (17)	0.0253 (17)	0.0278 (19)	−0.0005 (13)	0.0023 (14)	0.0027 (14)
C31	0.0176 (17)	0.0211 (15)	0.0185 (16)	0.0017 (13)	0.0020 (13)	0.0025 (12)
C32	0.0099 (15)	0.0143 (13)	0.0198 (16)	0.0036 (11)	0.0040 (12)	0.0002 (11)
C33	0.0166 (17)	0.0125 (14)	0.035 (2)	0.0006 (12)	0.0024 (15)	0.0020 (13)
C34	0.0181 (17)	0.0146 (15)	0.037 (2)	0.0028 (13)	0.0040 (15)	0.0050 (13)
C35	0.0125 (16)	0.0246 (17)	0.0308 (19)	0.0044 (13)	0.0027 (14)	0.0079 (14)
C36	0.0103 (15)	0.0215 (16)	0.0247 (17)	−0.0006 (12)	0.0038 (13)	0.0051 (13)
C37	0.0160 (16)	0.0120 (13)	0.0178 (15)	0.0007 (11)	0.0033 (12)	0.0010 (11)
C38	0.0158 (16)	0.0129 (14)	0.0226 (17)	−0.0018 (12)	0.0005 (13)	−0.0006 (12)
C39	0.0142 (16)	0.0226 (16)	0.0173 (16)	0.0018 (12)	0.0007 (13)	−0.0055 (12)
C40	0.024 (2)	0.025 (2)	0.096 (4)	0.0047 (17)	−0.014 (2)	−0.020 (2)
C41	0.029 (2)	0.029 (2)	0.080 (4)	0.0149 (18)	−0.018 (2)	−0.025 (2)
C42	0.0180 (19)	0.052 (3)	0.031 (2)	0.0180 (17)	−0.0022 (16)	−0.0054 (18)
C43	0.0119 (18)	0.046 (2)	0.051 (3)	−0.0042 (16)	−0.0009 (17)	0.013 (2)
C44	0.0205 (18)	0.0286 (18)	0.0257 (19)	0.0018 (14)	0.0001 (15)	0.0091 (14)
C45	0.018 (2)	0.012 (3)	0.007 (3)	0.0003 (18)	0.000 (2)	0.004 (3)
C46	0.019 (3)	0.026 (4)	0.011 (3)	−0.006 (2)	0.003 (2)	0.000 (2)
C47	0.016 (4)	0.026 (3)	0.014 (3)	−0.005 (2)	0.002 (2)	−0.002 (2)
C48	0.023 (4)	0.029 (3)	0.015 (3)	−0.001 (2)	0.002 (2)	−0.008 (2)
C49	0.028 (3)	0.036 (3)	0.020 (3)	0.004 (3)	0.006 (2)	−0.012 (2)
C50	0.020 (2)	0.026 (3)	0.017 (3)	−0.001 (2)	0.0026 (19)	−0.006 (2)
C51	0.018 (2)	0.0112 (13)	0.0113 (15)	0.0006 (15)	−0.0003 (14)	−0.0019 (11)
C52	0.019 (3)	0.039 (3)	0.017 (2)	−0.004 (2)	0.006 (2)	−0.012 (2)
C53	0.037 (3)	0.041 (3)	0.017 (3)	−0.006 (2)	0.016 (2)	−0.012 (2)
C54	0.031 (6)	0.026 (3)	0.0171 (19)	0.008 (5)	−0.002 (4)	−0.010 (2)
C55	0.042 (3)	0.021 (3)	0.020 (3)	−0.011 (3)	−0.007 (3)	−0.003 (2)
C56	0.033 (3)	0.024 (3)	0.019 (3)	−0.006 (3)	0.001 (2)	−0.001 (2)
C45B	0.018 (2)	0.012 (3)	0.007 (3)	0.0003 (18)	0.000 (2)	0.004 (3)
C46B	0.019 (3)	0.026 (4)	0.011 (3)	−0.006 (2)	0.003 (2)	0.000 (2)
C47B	0.016 (4)	0.026 (3)	0.014 (3)	−0.005 (2)	0.002 (2)	−0.002 (2)
C48B	0.023 (4)	0.029 (3)	0.015 (3)	−0.001 (2)	0.002 (2)	−0.008 (2)
C49B	0.028 (3)	0.036 (3)	0.020 (3)	0.004 (3)	0.006 (2)	−0.012 (2)
C50B	0.020 (2)	0.026 (3)	0.017 (3)	−0.001 (2)	0.0026 (19)	−0.006 (2)
C51B	0.018 (2)	0.0112 (13)	0.0113 (15)	0.0006 (15)	−0.0003 (14)	−0.0019 (11)
C52B	0.019 (3)	0.039 (3)	0.017 (2)	−0.004 (2)	0.006 (2)	−0.012 (2)
C53B	0.037 (3)	0.041 (3)	0.017 (3)	−0.006 (2)	0.016 (2)	−0.012 (2)
C54B	0.031 (6)	0.026 (3)	0.0171 (19)	0.008 (5)	−0.002 (4)	−0.010 (2)
C55B	0.042 (3)	0.021 (3)	0.020 (3)	−0.011 (3)	−0.007 (3)	−0.003 (2)
C56B	0.033 (3)	0.024 (3)	0.019 (3)	−0.006 (3)	0.001 (2)	−0.001 (2)
C57	0.0147 (15)	0.0159 (13)	0.0120 (14)	0.0002 (12)	0.0058 (12)	−0.0022 (11)
C58	0.0162 (16)	0.0141 (14)	0.0177 (16)	0.0019 (12)	0.0022 (13)	−0.0028 (11)
C59	0.0147 (16)	0.0255 (16)	0.0198 (17)	0.0018 (13)	−0.0020 (13)	−0.0056 (13)
C60	0.0114 (16)	0.0250 (16)	0.0246 (18)	−0.0059 (13)	0.0053 (13)	−0.0084 (13)
C61	0.0222 (17)	0.0141 (14)	0.0180 (16)	−0.0032 (12)	0.0088 (13)	−0.0026 (11)
C62	0.0129 (15)	0.0179 (14)	0.0122 (14)	0.0027 (11)	0.0029 (12)	−0.0007 (11)
C63	0.0131 (15)	0.0132 (13)	0.0104 (14)	0.0013 (11)	−0.0006 (11)	−0.0031 (10)
C64	0.0076 (14)	0.0114 (12)	0.0112 (14)	−0.0009 (10)	0.0038 (11)	−0.0010 (10)
C65	0.0114 (15)	0.0175 (14)	0.0106 (14)	0.0013 (11)	0.0027 (11)	0.0031 (11)
C66	0.0117 (15)	0.0243 (16)	0.0112 (14)	−0.0027 (12)	−0.0012 (12)	−0.0010 (11)
C67	0.0147 (16)	0.0168 (14)	0.0219 (17)	−0.0036 (12)	0.0032 (13)	−0.0013 (12)
C68	0.0166 (17)	0.0153 (14)	0.0269 (18)	−0.0028 (12)	0.0024 (14)	0.0073 (12)
C69	0.0138 (16)	0.0165 (14)	0.0185 (16)	0.0020 (12)	−0.0002 (12)	0.0037 (11)
C70	0.0087 (14)	0.0115 (12)	0.0123 (14)	0.0001 (10)	−0.0010 (11)	−0.0001 (10)
C71	0.0153 (15)	0.0166 (14)	0.0105 (14)	−0.0018 (12)	0.0023 (12)	0.0006 (11)
C72	0.0118 (15)	0.0170 (14)	0.0157 (15)	−0.0015 (11)	−0.0027 (12)	0.0020 (11)
C73	0.0107 (15)	0.0121 (13)	0.0246 (17)	−0.0012 (11)	−0.0024 (13)	0.0025 (12)
C74	0.0158 (16)	0.0168 (14)	0.0163 (15)	−0.0011 (12)	0.0071 (13)	−0.0022 (11)
C75	0.0121 (15)	0.0125 (13)	0.0160 (15)	−0.0002 (11)	0.0018 (12)	−0.0013 (11)
C76	0.031 (2)	0.0252 (17)	0.0129 (16)	0.0021 (14)	0.0050 (14)	0.0115 (13)
C77	0.0220 (18)	0.0207 (15)	0.0173 (16)	−0.0021 (13)	0.0039 (13)	0.0095 (12)
C78	0.0222 (18)	0.0271 (18)	0.0232 (18)	−0.0030 (14)	0.0023 (15)	0.0037 (14)
C79	0.0204 (19)	0.035 (2)	0.031 (2)	−0.0046 (15)	0.0029 (16)	0.0097 (16)
C80	0.027 (2)	0.0289 (19)	0.034 (2)	0.0049 (15)	0.0104 (17)	0.0097 (15)
C81	0.038 (2)	0.0239 (17)	0.0246 (19)	−0.0007 (16)	0.0050 (17)	0.0048 (14)
C82	0.0273 (19)	0.0233 (16)	0.0174 (17)	−0.0005 (14)	−0.0004 (14)	0.0045 (13)

### Geometric parameters (Å, º)

**Table:**

Ag1—P6	2.4548 (9)	C36—C37	1.395 (4)
Ag1—P1	2.4586 (9)	C36—H36	0.9500
Ag1—Br1	2.8314 (6)	C37—H37	0.9500
Ag1—Br2	2.8888 (6)	C38—H38A	0.9900
Ag1—Ag2	3.1046 (6)	C38—H38B	0.9900
Ag1—Ag3	3.3556 (6)	C39—C40	1.375 (5)
N1—C76	1.342 (4)	C39—C44	1.378 (5)
N1—C77	1.422 (4)	C40—C41	1.385 (6)
N1—H1	0.8800	C40—H40	0.9500
Ag2—P2	2.4402 (9)	C41—C42	1.383 (6)
Ag2—P3	2.4420 (9)	C41—H41	0.9500
Ag2—Br1	2.7543 (6)	C42—C43	1.367 (6)
Ag2—Br2	2.8226 (6)	C42—H42	0.9500
Ag2—Ag3	3.2336 (7)	C43—C44	1.397 (5)
N2—C76	1.360 (4)	C43—H43	0.9500
N2—H2A	0.8800	C44—H44	0.9500
N2—H2B	0.8800	C45—C46	1.386 (10)
Ag3—P5	2.4372 (9)	C45—C50	1.389 (10)
Ag3—P4	2.4405 (10)	C46—C47	1.391 (9)
Ag3—Br2	2.8230 (7)	C46—H46	0.9500
Ag3—Br1	2.8301 (6)	C47—C48	1.354 (9)
S1—C76	1.679 (4)	C47—H47	0.9500
P1—C7	1.825 (3)	C48—C49	1.390 (9)
P1—C1	1.826 (3)	C48—H48	0.9500
P1—C13	1.841 (3)	C49—C50	1.388 (8)
P2—C14	1.814 (3)	C49—H49	0.9500
P2—C20	1.825 (3)	C50—H50	0.9500
P2—C13	1.827 (3)	C51—C52	1.381 (12)
P3—C32	1.807 (3)	C51—C56	1.390 (11)
P3—C26	1.821 (3)	C52—C53	1.404 (8)
P3—C38	1.835 (3)	C52—H52	0.9500
P4—C45B	1.771 (13)	C53—C54	1.373 (12)
P4—C39	1.824 (3)	C53—H53	0.9500
P4—C38	1.835 (3)	C54—C55	1.380 (13)
P4—C45	1.887 (11)	C54—H54	0.9500
P5—C51	1.813 (12)	C55—C56	1.395 (8)
P5—C57	1.814 (3)	C55—H55	0.9500
P5—C63	1.840 (3)	C56—H56	0.9500
P5—C51B	1.852 (12)	C45B—C50B	1.387 (11)
P6—C70	1.823 (3)	C45B—C46B	1.395 (11)
P6—C64	1.824 (3)	C46B—C47B	1.394 (10)
P6—C63	1.846 (3)	C46B—H46B	0.9500
C1—C2	1.396 (4)	C47B—C48B	1.359 (10)
C1—C6	1.401 (4)	C47B—H47B	0.9500
C2—C3	1.392 (4)	C48B—C49B	1.408 (10)
C2—H2	0.9500	C48B—H48B	0.9500
C3—C4	1.388 (5)	C49B—C50B	1.387 (9)
C3—H3	0.9500	C49B—H49B	0.9500
C4—C5	1.380 (5)	C50B—H50B	0.9500
C4—H4	0.9500	C51B—C52B	1.381 (12)
C5—C6	1.387 (4)	C51B—C56B	1.391 (12)
C5—H5	0.9500	C52B—C53B	1.395 (8)
C6—H6	0.9500	C52B—H52B	0.9500
C7—C12	1.391 (4)	C53B—C54B	1.364 (12)
C7—C8	1.396 (4)	C53B—H53B	0.9500
C8—C9	1.389 (5)	C54B—C55B	1.368 (11)
C8—H8	0.9500	C54B—H54B	0.9500
C9—C10	1.386 (5)	C55B—C56B	1.414 (9)
C9—H9	0.9500	C55B—H55B	0.9500
C10—C11	1.392 (5)	C56B—H56B	0.9500
C10—H10	0.9500	C57—C62	1.397 (4)
C11—C12	1.382 (5)	C57—C58	1.397 (4)
C11—H11	0.9500	C58—C59	1.395 (4)
C12—H12	0.9500	C58—H58	0.9500
C13—H13A	0.9900	C59—C60	1.384 (5)
C13—H13B	0.9900	C59—H59	0.9500
C14—C19	1.396 (4)	C60—C61	1.384 (5)
C14—C15	1.399 (4)	C60—H60	0.9500
C15—C16	1.386 (4)	C61—C62	1.383 (4)
C15—H15	0.9500	C61—H61	0.9500
C16—C17	1.389 (4)	C62—H62	0.9500
C16—H16	0.9500	C63—H63A	0.9900
C17—C18	1.380 (5)	C63—H63B	0.9900
C17—H17	0.9500	C64—C69	1.393 (4)
C18—C19	1.386 (5)	C64—C65	1.400 (4)
C18—H18	0.9500	C65—C66	1.388 (4)
C19—H19	0.9500	C65—H65	0.9500
C20—C25	1.389 (4)	C66—C67	1.378 (4)
C20—C21	1.396 (4)	C66—H66	0.9500
C21—C22	1.391 (4)	C67—C68	1.385 (4)
C21—H21	0.9500	C67—H67	0.9500
C22—C23	1.377 (5)	C68—C69	1.389 (4)
C22—H22	0.9500	C68—H68	0.9500
C23—C24	1.388 (5)	C69—H69	0.9500
C23—H23	0.9500	C70—C71	1.397 (4)
C24—C25	1.387 (4)	C70—C75	1.402 (4)
C24—H24	0.9500	C71—C72	1.389 (4)
C25—H25	0.9500	C71—H71	0.9500
C26—C31	1.382 (4)	C72—C73	1.386 (4)
C26—C27	1.411 (4)	C72—H72	0.9500
C27—C28	1.377 (5)	C73—C74	1.395 (4)
C27—H27	0.9500	C73—H73	0.9500
C28—C29	1.385 (5)	C74—C75	1.388 (4)
C28—H28	0.9500	C74—H74	0.9500
C29—C30	1.379 (5)	C75—H75	0.9500
C29—H29	0.9500	C77—C82	1.388 (5)
C30—C31	1.394 (5)	C77—C78	1.396 (5)
C30—H30	0.9500	C78—C79	1.383 (5)
C31—H31	0.9500	C78—H78	0.9500
C32—C37	1.395 (4)	C79—C80	1.381 (5)
C32—C33	1.404 (4)	C79—H79	0.9500
C33—C34	1.378 (5)	C80—C81	1.390 (5)
C33—H33	0.9500	C80—H80	0.9500
C34—C35	1.389 (5)	C81—C82	1.383 (5)
C34—H34	0.9500	C81—H81	0.9500
C35—C36	1.370 (4)	C82—H82	0.9500
C35—H35	0.9500		
			
P6—Ag1—P1	125.44 (3)	C37—C32—C33	119.2 (3)
P6—Ag1—Br1	110.85 (2)	C37—C32—P3	119.5 (2)
P1—Ag1—Br1	105.31 (2)	C33—C32—P3	121.1 (2)
P6—Ag1—Br2	99.60 (2)	C34—C33—C32	120.7 (3)
P1—Ag1—Br2	116.14 (2)	C34—C33—H33	119.6
Br1—Ag1—Br2	95.646 (19)	C32—C33—H33	119.6
P6—Ag1—Ag2	145.149 (19)	C33—C34—C35	119.2 (3)
P1—Ag1—Ag2	89.24 (2)	C33—C34—H34	120.4
Br1—Ag1—Ag2	55.059 (11)	C35—C34—H34	120.4
Br2—Ag1—Ag2	56.052 (15)	C36—C35—C34	121.1 (3)
P6—Ag1—Ag3	85.79 (2)	C36—C35—H35	119.4
P1—Ag1—Ag3	148.603 (19)	C34—C35—H35	119.4
Br1—Ag1—Ag3	53.633 (11)	C35—C36—C37	120.2 (3)
Br2—Ag1—Ag3	53.117 (15)	C35—C36—H36	119.9
Ag2—Ag1—Ag3	59.922 (16)	C37—C36—H36	119.9
C76—N1—C77	130.7 (3)	C36—C37—C32	119.6 (3)
C76—N1—H1	114.6	C36—C37—H37	120.2
C77—N1—H1	114.6	C32—C37—H37	120.2
P2—Ag2—P3	118.29 (3)	P4—C38—P3	110.68 (16)
P2—Ag2—Br1	110.33 (2)	P4—C38—H38A	109.5
P3—Ag2—Br1	112.77 (2)	P3—C38—H38A	109.5
P2—Ag2—Br2	112.83 (3)	P4—C38—H38B	109.5
P3—Ag2—Br2	101.70 (2)	P3—C38—H38B	109.5
Br1—Ag2—Br2	98.946 (17)	H38A—C38—H38B	108.1
P2—Ag2—Ag1	89.64 (2)	C40—C39—C44	119.2 (3)
P3—Ag2—Ag1	151.34 (2)	C40—C39—P4	122.4 (3)
Br1—Ag2—Ag1	57.424 (11)	C44—C39—P4	118.3 (3)
Br2—Ag2—Ag1	58.103 (10)	C39—C40—C41	121.3 (4)
P2—Ag2—Ag3	153.52 (2)	C39—C40—H40	119.3
P3—Ag2—Ag3	88.11 (2)	C41—C40—H40	119.3
Br1—Ag2—Ag3	55.719 (9)	C42—C41—C40	119.1 (4)
Br2—Ag2—Ag3	55.063 (16)	C42—C41—H41	120.5
Ag1—Ag2—Ag3	63.895 (10)	C40—C41—H41	120.5
C76—N2—H2A	120.0	C43—C42—C41	120.3 (3)
C76—N2—H2B	120.0	C43—C42—H42	119.9
H2A—N2—H2B	120.0	C41—C42—H42	119.9
P5—Ag3—P4	129.41 (3)	C42—C43—C44	120.3 (4)
P5—Ag3—Br2	107.76 (2)	C42—C43—H43	119.9
P4—Ag3—Br2	99.89 (2)	C44—C43—H43	119.9
P5—Ag3—Br1	105.49 (2)	C39—C44—C43	119.9 (3)
P4—Ag3—Br1	112.15 (2)	C39—C44—H44	120.1
Br2—Ag3—Br1	97.161 (15)	C43—C44—H44	120.1
P5—Ag3—Ag2	143.64 (2)	C46—C45—C50	118.8 (9)
P4—Ag3—Ag2	86.82 (2)	C46—C45—P4	122.9 (6)
Br2—Ag3—Ag2	55.050 (9)	C50—C45—P4	118.3 (8)
Br1—Ag3—Ag2	53.529 (15)	C45—C46—C47	120.2 (8)
P5—Ag3—Ag1	87.50 (2)	C45—C46—H46	119.9
P4—Ag3—Ag1	142.31 (2)	C47—C46—H46	119.9
Br2—Ag3—Ag1	54.935 (9)	C48—C47—C46	120.7 (7)
Br1—Ag3—Ag1	53.667 (12)	C48—C47—H47	119.7
Ag2—Ag3—Ag1	56.183 (10)	C46—C47—H47	119.7
Ag2—Br1—Ag3	70.752 (15)	C47—C48—C49	120.2 (7)
Ag2—Br1—Ag1	67.518 (17)	C47—C48—H48	119.9
Ag3—Br1—Ag1	72.699 (14)	C49—C48—H48	119.9
Ag2—Br2—Ag3	69.888 (16)	C50—C49—C48	119.4 (7)
Ag2—Br2—Ag1	65.845 (13)	C50—C49—H49	120.3
Ag3—Br2—Ag1	71.948 (15)	C48—C49—H49	120.3
C7—P1—C1	108.24 (14)	C49—C50—C45	120.6 (7)
C7—P1—C13	104.26 (13)	C49—C50—H50	119.7
C1—P1—C13	101.13 (13)	C45—C50—H50	119.7
C7—P1—Ag1	113.54 (10)	C52—C51—C56	118.5 (9)
C1—P1—Ag1	113.32 (10)	C52—C51—P5	122.0 (8)
C13—P1—Ag1	115.24 (10)	C56—C51—P5	119.3 (8)
C14—P2—C20	106.61 (14)	C51—C52—C53	120.9 (7)
C14—P2—C13	106.97 (14)	C51—C52—H52	119.6
C20—P2—C13	101.68 (13)	C53—C52—H52	119.6
C14—P2—Ag2	115.97 (10)	C54—C53—C52	119.8 (7)
C20—P2—Ag2	110.90 (10)	C54—C53—H53	120.1
C13—P2—Ag2	113.54 (10)	C52—C53—H53	120.1
C32—P3—C26	101.34 (14)	C53—C54—C55	120.2 (9)
C32—P3—C38	105.31 (14)	C53—C54—H54	119.9
C26—P3—C38	105.82 (15)	C55—C54—H54	119.9
C32—P3—Ag2	121.31 (10)	C54—C55—C56	119.8 (7)
C26—P3—Ag2	107.94 (10)	C54—C55—H55	120.1
C38—P3—Ag2	113.52 (10)	C56—C55—H55	120.1
C45B—P4—C39	102.0 (4)	C51—C56—C55	120.8 (8)
C45B—P4—C38	98.8 (4)	C51—C56—H56	119.6
C39—P4—C38	105.94 (15)	C55—C56—H56	119.6
C39—P4—C45	102.2 (4)	C50B—C45B—C46B	119.5 (9)
C38—P4—C45	107.7 (3)	C50B—C45B—P4	119.5 (8)
C45B—P4—Ag3	114.1 (4)	C46B—C45B—P4	120.3 (8)
C39—P4—Ag3	118.08 (11)	C47B—C46B—C45B	120.5 (8)
C38—P4—Ag3	115.44 (10)	C47B—C46B—H46B	119.7
C45—P4—Ag3	106.3 (4)	C45B—C46B—H46B	119.7
C51—P5—C57	106.5 (3)	C48B—C47B—C46B	119.7 (8)
C51—P5—C63	100.6 (5)	C48B—C47B—H47B	120.2
C57—P5—C63	105.28 (14)	C46B—C47B—H47B	120.2
C57—P5—C51B	102.6 (3)	C47B—C48B—C49B	120.6 (8)
C63—P5—C51B	102.7 (6)	C47B—C48B—H48B	119.7
C51—P5—Ag3	115.4 (5)	C49B—C48B—H48B	119.7
C57—P5—Ag3	114.30 (10)	C50B—C49B—C48B	119.7 (7)
C63—P5—Ag3	113.44 (10)	C50B—C49B—H49B	120.1
C51B—P5—Ag3	116.9 (5)	C48B—C49B—H49B	120.1
C70—P6—C64	103.72 (13)	C49B—C50B—C45B	119.9 (8)
C70—P6—C63	103.13 (13)	C49B—C50B—H50B	120.1
C64—P6—C63	106.04 (13)	C45B—C50B—H50B	120.1
C70—P6—Ag1	111.71 (10)	C52B—C51B—C56B	119.2 (10)
C64—P6—Ag1	112.57 (9)	C52B—C51B—P5	122.7 (8)
C63—P6—Ag1	118.29 (10)	C56B—C51B—P5	118.0 (8)
C2—C1—C6	118.5 (3)	C51B—C52B—C53B	120.7 (8)
C2—C1—P1	117.9 (2)	C51B—C52B—H52B	119.7
C6—C1—P1	123.2 (2)	C53B—C52B—H52B	119.7
C3—C2—C1	120.8 (3)	C54B—C53B—C52B	119.9 (8)
C3—C2—H2	119.6	C54B—C53B—H53B	120.0
C1—C2—H2	119.6	C52B—C53B—H53B	120.0
C4—C3—C2	119.9 (3)	C53B—C54B—C55B	120.8 (10)
C4—C3—H3	120.1	C53B—C54B—H54B	119.6
C2—C3—H3	120.1	C55B—C54B—H54B	119.6
C5—C4—C3	119.8 (3)	C54B—C55B—C56B	119.8 (9)
C5—C4—H4	120.1	C54B—C55B—H55B	120.1
C3—C4—H4	120.1	C56B—C55B—H55B	120.1
C4—C5—C6	120.6 (3)	C51B—C56B—C55B	119.5 (8)
C4—C5—H5	119.7	C51B—C56B—H56B	120.2
C6—C5—H5	119.7	C55B—C56B—H56B	120.2
C5—C6—C1	120.3 (3)	C62—C57—C58	119.2 (3)
C5—C6—H6	119.9	C62—C57—P5	123.0 (2)
C1—C6—H6	119.9	C58—C57—P5	117.8 (2)
C12—C7—C8	119.5 (3)	C59—C58—C57	119.9 (3)
C12—C7—P1	117.2 (2)	C59—C58—H58	120.0
C8—C7—P1	123.2 (2)	C57—C58—H58	120.0
C9—C8—C7	120.0 (3)	C60—C59—C58	120.2 (3)
C9—C8—H8	120.0	C60—C59—H59	119.9
C7—C8—H8	120.0	C58—C59—H59	119.9
C10—C9—C8	119.9 (3)	C61—C60—C59	119.9 (3)
C10—C9—H9	120.1	C61—C60—H60	120.0
C8—C9—H9	120.1	C59—C60—H60	120.0
C9—C10—C11	120.4 (3)	C62—C61—C60	120.4 (3)
C9—C10—H10	119.8	C62—C61—H61	119.8
C11—C10—H10	119.8	C60—C61—H61	119.8
C12—C11—C10	119.7 (3)	C61—C62—C57	120.3 (3)
C12—C11—H11	120.2	C61—C62—H62	119.9
C10—C11—H11	120.2	C57—C62—H62	119.9
C11—C12—C7	120.6 (3)	P5—C63—P6	113.95 (15)
C11—C12—H12	119.7	P5—C63—H63A	108.8
C7—C12—H12	119.7	P6—C63—H63A	108.8
P2—C13—P1	113.37 (15)	P5—C63—H63B	108.8
P2—C13—H13A	108.9	P6—C63—H63B	108.8
P1—C13—H13A	108.9	H63A—C63—H63B	107.7
P2—C13—H13B	108.9	C69—C64—C65	119.2 (3)
P1—C13—H13B	108.9	C69—C64—P6	123.0 (2)
H13A—C13—H13B	107.7	C65—C64—P6	117.8 (2)
C19—C14—C15	119.0 (3)	C66—C65—C64	120.1 (3)
C19—C14—P2	116.8 (2)	C66—C65—H65	119.9
C15—C14—P2	124.1 (2)	C64—C65—H65	119.9
C16—C15—C14	120.2 (3)	C67—C66—C65	120.1 (3)
C16—C15—H15	119.9	C67—C66—H66	119.9
C14—C15—H15	119.9	C65—C66—H66	119.9
C15—C16—C17	120.0 (3)	C66—C67—C68	120.4 (3)
C15—C16—H16	120.0	C66—C67—H67	119.8
C17—C16—H16	120.0	C68—C67—H67	119.8
C18—C17—C16	120.2 (3)	C67—C68—C69	120.0 (3)
C18—C17—H17	119.9	C67—C68—H68	120.0
C16—C17—H17	119.9	C69—C68—H68	120.0
C17—C18—C19	120.0 (3)	C68—C69—C64	120.2 (3)
C17—C18—H18	120.0	C68—C69—H69	119.9
C19—C18—H18	120.0	C64—C69—H69	119.9
C18—C19—C14	120.5 (3)	C71—C70—C75	118.6 (3)
C18—C19—H19	119.7	C71—C70—P6	124.1 (2)
C14—C19—H19	119.7	C75—C70—P6	117.3 (2)
C25—C20—C21	119.2 (3)	C72—C71—C70	120.5 (3)
C25—C20—P2	116.5 (2)	C72—C71—H71	119.7
C21—C20—P2	124.2 (2)	C70—C71—H71	119.7
C22—C21—C20	119.7 (3)	C73—C72—C71	120.4 (3)
C22—C21—H21	120.1	C73—C72—H72	119.8
C20—C21—H21	120.1	C71—C72—H72	119.8
C23—C22—C21	120.7 (3)	C72—C73—C74	119.8 (3)
C23—C22—H22	119.6	C72—C73—H73	120.1
C21—C22—H22	119.6	C74—C73—H73	120.1
C22—C23—C24	119.8 (3)	C75—C74—C73	119.8 (3)
C22—C23—H23	120.1	C75—C74—H74	120.1
C24—C23—H23	120.1	C73—C74—H74	120.1
C25—C24—C23	119.9 (3)	C74—C75—C70	120.8 (3)
C25—C24—H24	120.0	C74—C75—H75	119.6
C23—C24—H24	120.0	C70—C75—H75	119.6
C24—C25—C20	120.6 (3)	N1—C76—N2	112.9 (3)
C24—C25—H25	119.7	N1—C76—S1	127.0 (3)
C20—C25—H25	119.7	N2—C76—S1	120.1 (3)
C31—C26—C27	118.4 (3)	C82—C77—C78	119.7 (3)
C31—C26—P3	124.4 (3)	C82—C77—N1	116.2 (3)
C27—C26—P3	116.9 (2)	C78—C77—N1	124.0 (3)
C28—C27—C26	120.5 (3)	C79—C78—C77	119.1 (3)
C28—C27—H27	119.7	C79—C78—H78	120.4
C26—C27—H27	119.7	C77—C78—H78	120.4
C27—C28—C29	120.2 (3)	C80—C79—C78	121.9 (4)
C27—C28—H28	119.9	C80—C79—H79	119.0
C29—C28—H28	119.9	C78—C79—H79	119.0
C30—C29—C28	120.1 (3)	C79—C80—C81	118.2 (4)
C30—C29—H29	119.9	C79—C80—H80	120.9
C28—C29—H29	119.9	C81—C80—H80	120.9
C29—C30—C31	119.9 (3)	C82—C81—C80	121.1 (3)
C29—C30—H30	120.1	C82—C81—H81	119.5
C31—C30—H30	120.1	C80—C81—H81	119.5
C26—C31—C30	120.8 (3)	C81—C82—C77	119.9 (3)
C26—C31—H31	119.6	C81—C82—H82	120.0
C30—C31—H31	119.6	C77—C82—H82	120.0
			
C7—P1—C1—C2	110.6 (3)	Ag3—P4—C45—C50	−74.2 (9)
C13—P1—C1—C2	−140.1 (3)	C50—C45—C46—C47	−2.6 (12)
Ag1—P1—C1—C2	−16.2 (3)	P4—C45—C46—C47	179.1 (8)
C7—P1—C1—C6	−76.9 (3)	C45—C46—C47—C48	0.6 (11)
C13—P1—C1—C6	32.3 (3)	C46—C47—C48—C49	2.6 (13)
Ag1—P1—C1—C6	156.2 (2)	C47—C48—C49—C50	−3.7 (13)
C6—C1—C2—C3	−2.6 (5)	C48—C49—C50—C45	1.7 (12)
P1—C1—C2—C3	170.2 (3)	C46—C45—C50—C49	1.5 (13)
C1—C2—C3—C4	1.0 (5)	P4—C45—C50—C49	179.8 (6)
C2—C3—C4—C5	1.4 (5)	C57—P5—C51—C52	27.9 (13)
C3—C4—C5—C6	−2.2 (5)	C63—P5—C51—C52	−81.7 (11)
C4—C5—C6—C1	0.5 (5)	C51B—P5—C51—C52	42 (15)
C2—C1—C6—C5	1.9 (5)	Ag3—P5—C51—C52	155.9 (9)
P1—C1—C6—C5	−170.6 (3)	C57—P5—C51—C56	−156.5 (10)
C1—P1—C7—C12	−155.8 (2)	C63—P5—C51—C56	93.9 (12)
C13—P1—C7—C12	97.1 (2)	C51B—P5—C51—C56	−143 (17)
Ag1—P1—C7—C12	−29.1 (3)	Ag3—P5—C51—C56	−28.5 (13)
C1—P1—C7—C8	28.4 (3)	C56—C51—C52—C53	0.4 (15)
C13—P1—C7—C8	−78.7 (3)	P5—C51—C52—C53	176.0 (9)
Ag1—P1—C7—C8	155.1 (2)	C51—C52—C53—C54	−0.2 (12)
C12—C7—C8—C9	−0.2 (4)	C52—C53—C54—C55	−0.1 (15)
P1—C7—C8—C9	175.5 (2)	C53—C54—C55—C56	0.1 (16)
C7—C8—C9—C10	−1.7 (5)	C52—C51—C56—C55	−0.3 (17)
C8—C9—C10—C11	2.5 (5)	P5—C51—C56—C55	−176.1 (8)
C9—C10—C11—C12	−1.3 (5)	C54—C55—C56—C51	0.1 (15)
C10—C11—C12—C7	−0.7 (5)	C39—P4—C45B—C50B	31.4 (10)
C8—C7—C12—C11	1.4 (4)	C38—P4—C45B—C50B	139.9 (9)
P1—C7—C12—C11	−174.5 (2)	C45—P4—C45B—C50B	−61 (4)
C14—P2—C13—P1	79.14 (19)	Ag3—P4—C45B—C50B	−97.0 (9)
C20—P2—C13—P1	−169.25 (16)	C39—P4—C45B—C46B	−158.4 (8)
Ag2—P2—C13—P1	−50.08 (18)	C38—P4—C45B—C46B	−49.9 (9)
C7—P1—C13—P2	−80.60 (18)	C45—P4—C45B—C46B	109 (5)
C1—P1—C13—P2	167.12 (16)	Ag3—P4—C45B—C46B	73.2 (9)
Ag1—P1—C13—P2	44.52 (19)	C50B—C45B—C46B—C47B	−2.3 (13)
C20—P2—C14—C19	108.6 (2)	P4—C45B—C46B—C47B	−172.5 (8)
C13—P2—C14—C19	−143.3 (2)	C45B—C46B—C47B—C48B	−0.2 (11)
Ag2—P2—C14—C19	−15.4 (3)	C46B—C47B—C48B—C49B	3.0 (14)
C20—P2—C14—C15	−75.0 (3)	C47B—C48B—C49B—C50B	−3.3 (14)
C13—P2—C14—C15	33.2 (3)	C48B—C49B—C50B—C45B	0.8 (13)
Ag2—P2—C14—C15	161.0 (2)	C46B—C45B—C50B—C49B	2.0 (14)
C19—C14—C15—C16	−0.3 (4)	P4—C45B—C50B—C49B	172.2 (7)
P2—C14—C15—C16	−176.6 (2)	C51—P5—C51B—C52B	175 (17)
C14—C15—C16—C17	0.6 (5)	C57—P5—C51B—C52B	−18.3 (13)
C15—C16—C17—C18	−0.9 (5)	C63—P5—C51B—C52B	−127.4 (11)
C16—C17—C18—C19	0.8 (5)	Ag3—P5—C51B—C52B	107.6 (10)
C17—C18—C19—C14	−0.5 (5)	C51—P5—C51B—C56B	−1 (14)
C15—C14—C19—C18	0.2 (5)	C57—P5—C51B—C56B	165.3 (11)
P2—C14—C19—C18	176.8 (2)	C63—P5—C51B—C56B	56.2 (12)
C14—P2—C20—C25	−155.1 (2)	Ag3—P5—C51B—C56B	−68.7 (13)
C13—P2—C20—C25	93.0 (2)	C56B—C51B—C52B—C53B	−0.6 (16)
Ag2—P2—C20—C25	−28.0 (3)	P5—C51B—C52B—C53B	−176.9 (10)
C14—P2—C20—C21	23.5 (3)	C51B—C52B—C53B—C54B	−0.6 (13)
C13—P2—C20—C21	−88.4 (3)	C52B—C53B—C54B—C55B	−0.2 (19)
Ag2—P2—C20—C21	150.5 (3)	C53B—C54B—C55B—C56B	2 (2)
C25—C20—C21—C22	1.4 (5)	C52B—C51B—C56B—C55B	2.5 (18)
P2—C20—C21—C22	−177.1 (3)	P5—C51B—C56B—C55B	179.1 (8)
C20—C21—C22—C23	0.2 (6)	C54B—C55B—C56B—C51B	−3.3 (16)
C21—C22—C23—C24	−1.7 (6)	C51—P5—C57—C62	−73.6 (6)
C22—C23—C24—C25	1.6 (5)	C63—P5—C57—C62	32.6 (3)
C23—C24—C25—C20	0.0 (5)	C51B—P5—C57—C62	−74.5 (6)
C21—C20—C25—C24	−1.6 (5)	Ag3—P5—C57—C62	157.8 (2)
P2—C20—C25—C24	177.1 (2)	C51—P5—C57—C58	107.0 (6)
C32—P3—C26—C31	136.6 (3)	C63—P5—C57—C58	−146.8 (2)
C38—P3—C26—C31	27.0 (3)	C51B—P5—C57—C58	106.0 (6)
Ag2—P3—C26—C31	−94.9 (3)	Ag3—P5—C57—C58	−21.7 (3)
C32—P3—C26—C27	−49.5 (3)	C62—C57—C58—C59	0.2 (4)
C38—P3—C26—C27	−159.2 (2)	P5—C57—C58—C59	179.7 (2)
Ag2—P3—C26—C27	79.0 (2)	C57—C58—C59—C60	−0.7 (5)
C31—C26—C27—C28	−1.2 (5)	C58—C59—C60—C61	0.2 (5)
P3—C26—C27—C28	−175.5 (2)	C59—C60—C61—C62	0.9 (5)
C26—C27—C28—C29	1.0 (5)	C60—C61—C62—C57	−1.4 (5)
C27—C28—C29—C30	0.0 (5)	C58—C57—C62—C61	0.9 (4)
C28—C29—C30—C31	−0.9 (5)	P5—C57—C62—C61	−178.6 (2)
C27—C26—C31—C30	0.4 (5)	C51—P5—C63—P6	−177.8 (4)
P3—C26—C31—C30	174.2 (2)	C57—P5—C63—P6	71.69 (19)
C29—C30—C31—C26	0.6 (5)	C51B—P5—C63—P6	178.8 (4)
C26—P3—C32—C37	130.7 (3)	Ag3—P5—C63—P6	−54.00 (18)
C38—P3—C32—C37	−119.2 (3)	C70—P6—C63—P5	169.06 (16)
Ag2—P3—C32—C37	11.4 (3)	C64—P6—C63—P5	−82.25 (18)
C26—P3—C32—C33	−44.3 (3)	Ag1—P6—C63—P5	45.22 (19)
C38—P3—C32—C33	65.8 (3)	C70—P6—C64—C69	30.3 (3)
Ag2—P3—C32—C33	−163.6 (2)	C63—P6—C64—C69	−77.9 (3)
C37—C32—C33—C34	−0.8 (5)	Ag1—P6—C64—C69	151.3 (2)
P3—C32—C33—C34	174.3 (3)	C70—P6—C64—C65	−147.9 (2)
C32—C33—C34—C35	0.3 (5)	C63—P6—C64—C65	103.8 (2)
C33—C34—C35—C36	0.3 (5)	Ag1—P6—C64—C65	−27.0 (2)
C34—C35—C36—C37	−0.5 (5)	C69—C64—C65—C66	−0.3 (4)
C35—C36—C37—C32	0.0 (5)	P6—C64—C65—C66	178.0 (2)
C33—C32—C37—C36	0.6 (5)	C64—C65—C66—C67	0.0 (5)
P3—C32—C37—C36	−174.5 (2)	C65—C66—C67—C68	0.5 (5)
C45B—P4—C38—P3	176.8 (5)	C66—C67—C68—C69	−0.5 (5)
C39—P4—C38—P3	−78.00 (18)	C67—C68—C69—C64	0.1 (5)
C45—P4—C38—P3	173.3 (4)	C65—C64—C69—C68	0.3 (4)
Ag3—P4—C38—P3	54.69 (18)	P6—C64—C69—C68	−177.9 (2)
C32—P3—C38—P4	79.67 (18)	C64—P6—C70—C71	−80.9 (3)
C26—P3—C38—P4	−173.50 (15)	C63—P6—C70—C71	29.5 (3)
Ag2—P3—C38—P4	−55.30 (18)	Ag1—P6—C70—C71	157.6 (2)
C45B—P4—C39—C40	48.8 (5)	C64—P6—C70—C75	98.2 (2)
C38—P4—C39—C40	−54.2 (4)	C63—P6—C70—C75	−151.4 (2)
C45—P4—C39—C40	58.5 (5)	Ag1—P6—C70—C75	−23.3 (2)
Ag3—P4—C39—C40	174.6 (3)	C75—C70—C71—C72	0.2 (4)
C45B—P4—C39—C44	−126.9 (5)	P6—C70—C71—C72	179.3 (2)
C38—P4—C39—C44	130.1 (3)	C70—C71—C72—C73	−0.3 (4)
C45—P4—C39—C44	−117.2 (4)	C71—C72—C73—C74	−0.4 (4)
Ag3—P4—C39—C44	−1.1 (3)	C72—C73—C74—C75	1.2 (4)
C44—C39—C40—C41	3.0 (7)	C73—C74—C75—C70	−1.3 (4)
P4—C39—C40—C41	−172.6 (4)	C71—C70—C75—C74	0.6 (4)
C39—C40—C41—C42	−1.3 (8)	P6—C70—C75—C74	−178.6 (2)
C40—C41—C42—C43	−0.8 (7)	C77—N1—C76—N2	−177.5 (3)
C41—C42—C43—C44	1.1 (7)	C77—N1—C76—S1	2.8 (5)
C40—C39—C44—C43	−2.7 (6)	C76—N1—C77—C82	−153.3 (3)
P4—C39—C44—C43	173.2 (3)	C76—N1—C77—C78	31.0 (5)
C42—C43—C44—C39	0.7 (6)	C82—C77—C78—C79	1.3 (5)
C45B—P4—C45—C46	−42 (4)	N1—C77—C78—C79	176.9 (3)
C39—P4—C45—C46	−131.6 (8)	C77—C78—C79—C80	−0.7 (5)
C38—P4—C45—C46	−20.3 (10)	C78—C79—C80—C81	−0.3 (5)
Ag3—P4—C45—C46	104.0 (8)	C79—C80—C81—C82	0.6 (5)
C45B—P4—C45—C50	140 (5)	C80—C81—C82—C77	0.1 (5)
C39—P4—C45—C50	50.1 (9)	C78—C77—C82—C81	−1.1 (5)
C38—P4—C45—C50	161.5 (7)	N1—C77—C82—C81	−176.9 (3)

### Hydrogen-bond geometry (Å, º)

**Table:**

*D*—H···*A*	*D*—H	H···*A*	*D*···*A*	*D*—H···*A*
N1—H1···Br3	0.88	2.46	3.328 (3)	167
N2—H2*A*···Br3	0.88	2.57	3.390 (3)	155
C6—H6···Br3^i^	0.95	2.93	3.855 (3)	166
C13—H13*A*···Br3^i^	0.99	2.80	3.705 (3)	152
C47—H47···S1^ii^	0.95	2.81	3.645 (9)	147
C78—H78···S1	0.95	2.70	3.259 (4)	118

Symmetry codes: (i) *x*+1/2, −*y*+1/2, *z*−1/2; (ii) −*x*+1, −*y*+1, −*z*+1.
