# Supplementary figures and images for: Crystal structure of tris­[μ2-bis­(di­phenyl­phosphan­yl)methane-κ2 P:P′]di-μ3-bromido-tris­ilver(I) bromide–N,N′-phenyl­thio­urea (1/1)
Source: Acta Crystallogr E Crystallogr Commun. 2015 Mar 21;71(Pt 4):m89–90. doi: 10.1107/S2056989015005150 (PMC4438794; doi:10.1107/S2056989015005150)

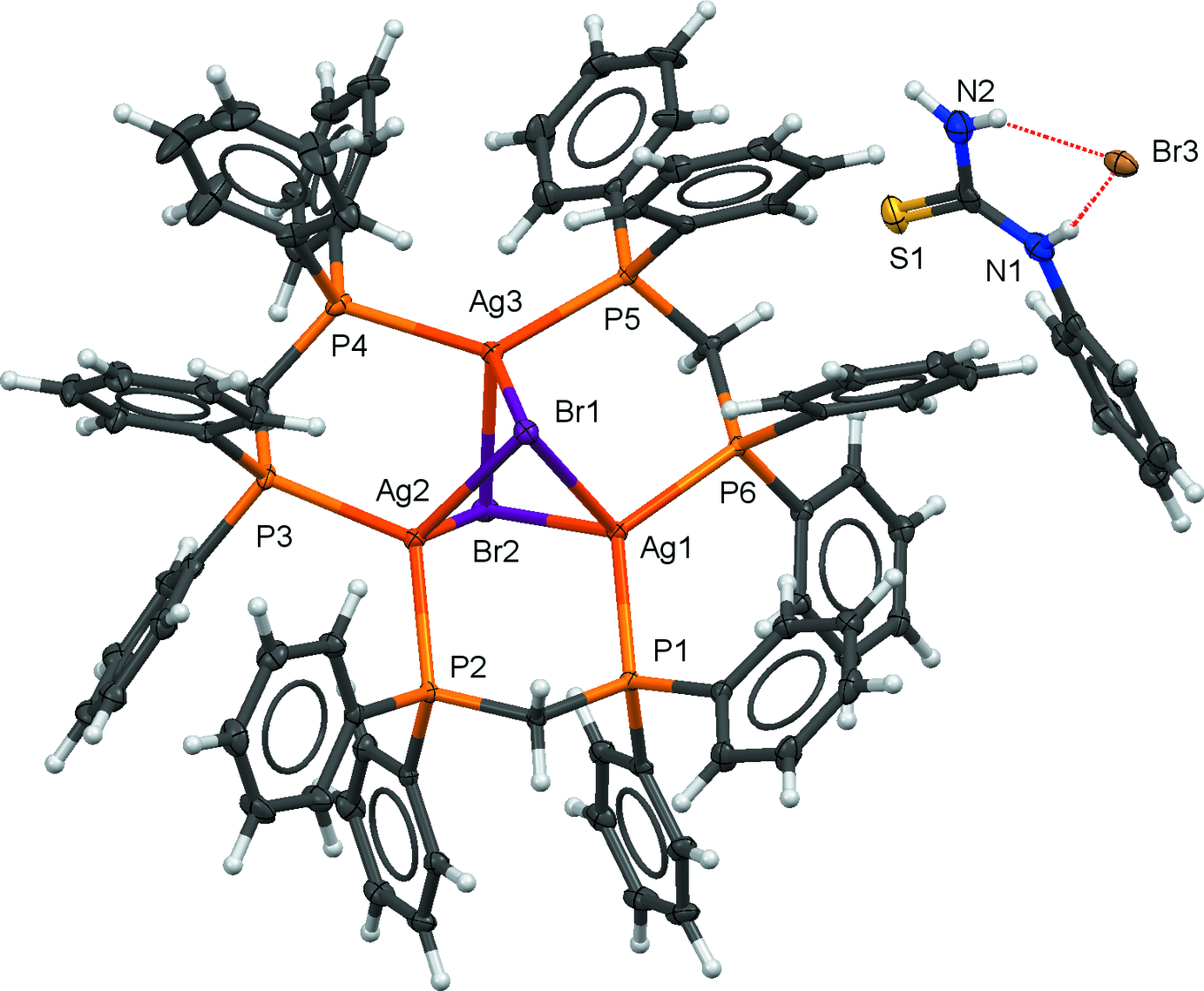

Supplement: Supplementary file 3 [file e-71-00m89-fig1.tif]

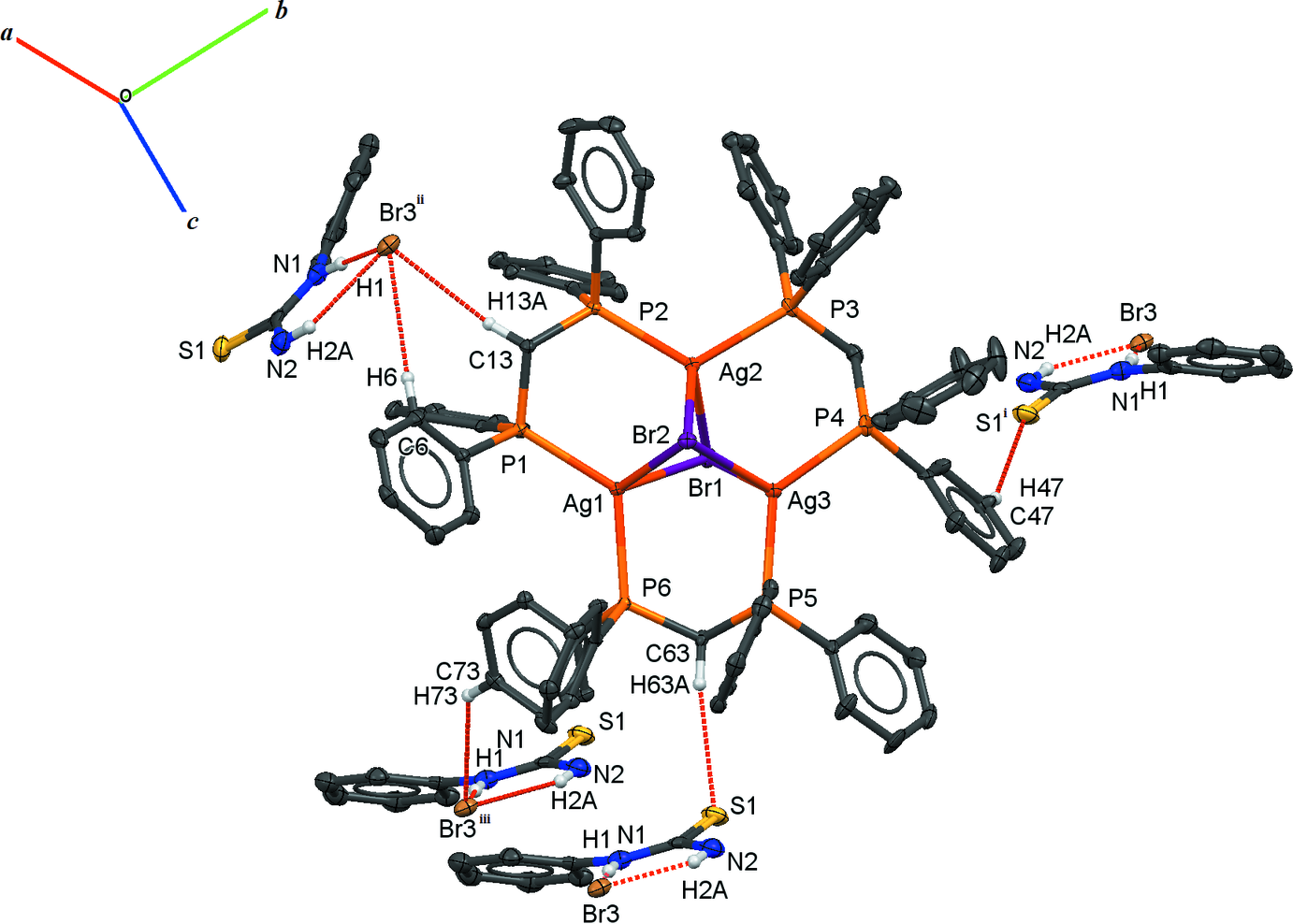

Supplement: Supplementary file 4 [file e-71-00m89-fig2.tif]
